# The relationship between 25-hydroxy vitamin D and serum asprosin in patients with type 2 diabetes in the community

**DOI:** 10.3389/fendo.2024.1409156

**Published:** 2024-07-31

**Authors:** Junfang Cui, Zhengqian Wang, Jianhong Yin, Mina Li, Qianqian Wu, Ming Liu, Hong Su, Huijuan Ren, Minggang Xu, Jing Yang, Linxin Xu

**Affiliations:** ^1^ Department of Endocrinology, First Hospital of Shanxi Medical University, Shanxi Medical University, Taiyuan, Shanxi, China; ^2^ First Clinical Medical College, Shanxi Medical University, Taiyuan, Shanxi, China; ^3^ Department of Endocrinology, Changzhi Second People's Hospital, Changzhi, Shanxi, China; ^4^ Shanxi Innovation Center for Integrated Management of Hypertension, Hyperlipidemia and Hyperglycemia Correlated with Cardiovascular and Cerebrovascular Diseases, Taiyuan, Shanxi, China

**Keywords:** diabetes, type 2, asprosin, 25-hydroxy vitamin D, community, insulin resistance

## Abstract

**Objectives:**

This study aimed to investigate the link between 25-hydroxy vitamin D and serum asprosin in individuals with type 2 diabetes within the community. The goal was to provide a foundation for clinical interventions.

**Methods:**

Between November 2019 and July 2021, data from 463 patients with type 2 diabetes were consistently gathered at a community health service station in Southeast Shanxi Province. General information and laboratory metrics were compiled, including serum asprosin levels. The participants were categorized based on three serum asprosin quantiles, allowing for a comparison of various factors among the groups. The correlation between serum asprosin levels and other factors was analyzed. Employing a general linear model, the connection between 25-hydroxy vitamin D and serum asprosin levels was studied. Utilizing three quantiles of 25-hydroxy vitamin D, serum asprosin was treated as the dependent variable, while 25-hydroxy vitamin D served as the independent variable for linear regression analysis.

**Results:**

As serum asprosin increased, there were gradual increments in age, disease duration, SBP, BMI, WC, creatinine, and SUA levels (P<0.05). Conversely, HbA1c, HDL-C, GFR, and 25-hydroxy vitamin D levels exhibited gradual declines (P<0.05). Age, 25-hydroxy vitamin D, SUA, creatinine, and LDL-C emerged as independent influencing factors for serum asprosin. Across the 1st to 3rd 25-hydroxy vitamin D quantiles, elevated 25-hydroxy vitamin D levels correlated with a gradual reduction in mean serum asprosin (P<0.05).

**Conclusion:**

Serum asprosin levels demonstrate an inverse correlation with 25-hydroxy vitamin D levels in community-dwelling individuals with type 2 diabetes. Serum asprosin levels might independently contribute to 25-hydroxy vitamin D levels.

## Introduction

In 2016, Romere et al. made the initial discovery of asprosin, encoded by two exons (exon 65 and exon 66) of the fibrillin 1 (FBN1) gene, as a novel adipokine protein involved in glucose production (1). This finding emerged from a study on patients with Neonatal Progeroid Syndrome (NPS). Individuals with NPS, who exhibit asprosin deficiency due to truncated FBN1 mutations, maintain normal blood glucose levels despite reduced plasma insulin levels (1). Extensive research has shown a relationship between asprosin concentration and insulin resistance (IR) as well as diabetes in both mice and humans. A cross-sectional analysis involving 143 subjects demonstrated elevated asprosin levels in Impaired Glucose Regulation (IGR) and Type 2 Diabetes Mellitus (T2DM) groups in comparison to the Normal Glucose Regulation (NGR) group. Asprosin exhibited positive correlation with insulin resistance homeostatic model assessment (HOMA-IR) and negative correlation with β-cell function (HOMA-β) (2). Moreover, a case-control study encompassing 170 subjects identified higher T2DM-associated asprosin levels in adults compared to the control group. Notably, an independent relationship between fasting plasma glucose and asprosin in T2DM patients was observed, in alignment with numerous other studies (3). Animal investigations have also indicated heightened liver asprosin levels in Type 1 Diabetes Mellitus (T1DM) mice (4). Consequently, serum asprosin could serve as a biomarker for early diabetes diagnosis.

The principal form of vitamin D in the body, 25-hydroxy vitamin D, contributes to preserving pancreatic islet β-cell function and inhibiting β-cell apoptosis (5). Studies have revealed that deficiency in 25-hydroxy vitamin D impacts glucose metabolism in individuals with Type 2 Diabetes Mellitus (T2DM) and is presumed to associate with insulin resistance (6). A link between adipocytokines and blood 25-hydroxy vitamin D levels has also been established. Among patients with T2DM, fasting serum asprosin levels increase and demonstrate a strong association with bone mineral density (3). Additionally, research involving prenatal screening for Trisomy 21 demonstrated an inverse correlation between amniotic fluid 25-hydroxy vitamin D and asprosin levels (7). Despite these findings, the intricate relationship among 25-hydroxy vitamin D, asprosin, and T2DM necessitates further elucidation. One plausible hypothesis is that 25-hydroxy vitamin D might influence the production and/or secretion of adipocytokines. Whether a linear negative correlation exists between these factors in T2DM patients remains uncertain. This study thus collected clinical data from community-based T2DM patients to analyze the connection between 25-hydroxy vitamin D and serum asprosin levels.

## Materials and methods

### Research participants

The subjects of the present report were those of the previous study (Xu et al, 2022) with the same inclusion criteria. Clinical and biochemical data were taken from the previous study (methods outlined in Xu et al, 2022) (8), possession of complete clinical data, and willingness to cooperate with the study. Exclusion criteria encompassed patients with renal insufficiency characterized by creatinine levels exceeding 178 μmol/L, type 1 diabetes, diabetes resulting from other endocrine disorders, diabetic ketoacidosis, diabetic hyperosmolar coma, severe liver or kidney dysfunction, severe infection, malignant tumors, individuals with communication disorders like mental illness, and those unable to collaborate with the study’s requirements. After excluding 35 participants due to incomplete clinical data, this investigation analyzed clinical information from 463 patients.

### Research methods

#### General data collection

General data collection involved measuring patients’ height and weight to calculate the body mass index (BMI). Patient demographic information, such as gender, age, abdominal circumference, systolic and diastolic blood pressure, and the usage of hypoglycemic medications and insulin, was gathered. Fasting venous blood samples were obtained to measure fasting plasma glucose (FPG), 2-hour postprandial plasma glucose (2hPG), serum uric acid (SUA), creatinine (CRE), total cholesterol (TC), triglyceride (TG), high-density lipoprotein-cholesterol (HDL-C), low-density lipoprotein-cholesterol (LDL-C), alanine aminotransferase (ALT), and aspartate aminotransferase (AST) using Beckman Automatic Biochemical Analyzer (USA, BK-200). High-pressure liquid phase assessment (Roche 501, Switzerland) was conducted to determine glycated hemoglobin A1c (HbA1c) levels. Roche Diagnostic Products (Shanghai) Co. Ltd provided serum 25-hydroxy vitamin D concentration determination kits, utilizing chemiluminescence methods with within-batch CV < 8% and between-batch CV < 10%.

#### Measurement of serum asprosin

After fasting for 8-10 hours, venous blood samples were collected the next morning, followed by centrifugation at 3000 rpm for 15 minutes. Serum asprosin levels were determined using enzyme-linked immunosorbent assay (ELISA) provided by Hepeng (Shanghai) Biotechnology Co. Ltd. All samples were stored at -80°C. Double-well duplication was employed for measurement, ensuring batch-to-batch difference <11% and intra-batch difference <8%. Strict adherence to kit and instrument manuals was maintained. Patients with T2DM were categorized into three groups based on serum asprosin tertiles: T1 (asprosin < 295.0 pg/ml, 153 cases), T2 (asprosin 295.0~370.5 pg/ml, 153 patients), and T3 (asprosin > 370.5 pg/ml, 157 cases).

#### Statistical processing

Statistical analysis was performed using SPSS 22.0 software. Normally distributed metric data were expressed as Mean ± SD. One-way ANOVA was used for group comparisons, followed by LSD tests for pairwise comparisons. Non-normally distributed measurement data were expressed as M(Q1, Q3), and Mann-Whitney rank sum tests were applied for intergroup comparisons. Pearson correlation analysis was conducted for normally distributed data, while Spearman correlation analysis was used for data not conforming to bivariate normal distribution. The relationship between serum asprosin and 25-hydroxyvitamin D tertiles was evaluated using a general linear model, with statistical significance defined as P < 0.05.

## Results

### The clinical characteristics of the patient

Among the 463 patients with type 2 diabetes, 266 were male with a mean age of 54.3 ± 13.0 years, and 197 were female with a mean age of 59.6 ± 12.1 years. The median duration of diabetes was 10 years, and the average glycated hemoglobin was 8.8%. Patients were categorized into three groups based on serum asprosin quantiles. As serum asprosin levels increased, patient age, disease duration, SBP, BMI, WC, creatinine, and SUA levels also increased, while levels of HbA1c, HDL-C, GFR, and 25-hydroxy vitamin D decreased (**Table 1**).

Table 1Comparison of clinical data for 463 community-based patients with type 2 diabetes.

**Table:** 

Serum asprosin (pg/ml)	Gender (male /female, e.g)	Course of disease* (years)	Age (years)	SBP (mmHg)	DBP (mmHg)	BMI (kg/m^2^)	WC (cm)	TC (mmol/L)	TG* (mmol/L)	LDL-C (mmol/L)
<295	153(71/82)	9.0 (4.0,15.5)	53.5 ± 13.1	130.1 ± 17.4	78.8 ± 10.4	25.1 ± 3.6	90.78 ± 10.56	4.6 ± 1.1	1.5 (1.0,2.2)	2.7 ± 0.8
295∼370.5	156(96/60)	10.0 (5.0,16.0)	57.3 ± 12.5	132.5 ± 17.3	79.8 ± 10.9	25.8 ± 3.7	93.32 ± 10.06	4.7 ± 1.2	1.5 (1.1,2.1)	2.8 ± 1.0
>370.5	154(99/55)	12.0 (16.0.17.0)	58.8 ± 12.5	135.5 ± 17.8	80.4 ± 10.7	26.8 ± 4.0	98.26 ± 11.35	4.6 ± 1.2	1.7 (1.2,2.5)	2.7 ± 0.8
χ2/F value	6.099	5.984	7.138	3.700	0.885	8.554	19.278	0.404	6.163	1.688
*P-value*	0.002	0.050	0.001	0.025	0.413	<0.001	<0.001	0.668	0.046	0.186

**Table:** 

Serum asprosin (pg/ml)	HDL-C (mmol/L)	AST* (mmol/L)	ALT* (mmol/L)	CRE* (µmol/L)	GFR (mL/min)	FPG (mmol/L)	PBG (mmol/L)	HbA1c (%)	SUA (µmol/L)	25-hydroxy vitamin D (nmol/L)
<295	1.0 ± 0.3	17.0 (14.0,21.5)	16.0 (12.5,26.5)	56.0 (46.0,66.0)	126.77 ± 35.61	8.2 ± 3.0	13.2 ± 4.2	9.3 ± 2.1	262.4 ± 60.8	31.0 (25.5,39.4)
295∼370.5	0.98 ± 0.25	18.0 (15.0, 24.0)	17.0 (13.0,29.0)	63.0 (52.0,72.5)	113.34 ± 31.44	7.5 ± 2.8	12.0 ± 3.9	8.7 ± 1.8	329.6 ± 54.3	30.7 (24.9,37.6)
>370.5	0.9 ± 0.2	20.0 (16.0,27.0)	22.0 (14.0,34.0)	68.0 (58.0,80.0)	107.31 ± 67.26	7.5 ± 2.4	12.1 ± 4.0	8.4 ± 1.7	392.7 ± 83.0	29.2 (21.6,37.5)
χ2/z/t value	3.421	2.915	1.680	51.195	6.726	3.857	3.996	9.224	145.285	3.542
*P-value*	0.034	0.055	0.188	<0.001	0.001	0.022	0.019	<0.001	<0.001	0.030

**Table:** 

Serum asprosin (pg/ml)	taking metformin (n,%)	taking glycosidase inhibitors (n,%)	taking sulfonylureas (n,%)	taking DPPIV enzyme inhibitors (n,%)	taking thiazolidinedione (n,%)	using insulin (n,%)
<295	78 (51.0)	42(27.5)	34(22.2)	0(0)	2(1.3)	69(45.1)
295∼370.5	69(45.1)	47(30.7)	33(21.6)	3(2.0)	7(4.6)	82(53.6)
>370.5	77(49.0)	58(36.9)	39(24.8)	4(2.5)	1(0.6)	77(49)
χ2/z/t value	1.061	1.130	0.869	0.856	0.450	1.060
*P-value*	0.328	0.178	0.857	0.881	1.000	0.331

Continuous data are presented as mean ± standard deviation. Count data are presented as percentages. Skewed distribution data are expressed as median (25th, 75th) percentiles.

SBP, Systolic Blood Pressure; DBP, Diastolic Blood Pressure; BMI, Body Mass Index; WC, Waist Circumference; TC, Total Cholesterol; TG, Triglycerides; LDL-C, Low-Density Lipoprotein Cholesterol; HDL-C, High-Density Lipoprotein Cholesterol; AST, Aspartate Aminotransferase; ALT, Alanine Aminotransferase; CRE, Serum Creatinine; GFR, Glomerular Filtration Rate; FPG, Fasting Plasma Glucose; PBG, 2-Hour Postprandial Blood Glucose; HbA1c, Glycated Hemoglobin; SUA, Serum Uric Acid.

*Means non normal distribution.

### The correlation between serum asprosin and other variables

Serum asprosin displayed positive correlations with disease duration, age, SBP, BMI, WC, TG, creatinine, SUA, AST, and ALT (r = 0.114, 0.168, 0.126, 0.189, 0.276, 0.104, 0.332, 0.622, 0.205, and 0.144, respectively; P < 0.05). Conversely, serum asprosin exhibited negative correlations with HDL-C, GFR, FPG, HbA1c, and 25-hydroxy vitamin D (r = -0.121, -0.165, -0.113, -0.194, and -0.122, respectively; P < 0.05) (**Table 2**).

**Table 2:** Correlation analysis results between serum asprosin and other variables.

variable	Groups	
	*r*	*P*
Duration of the disease* (years)	0.114	0.015
Age (years)	0.168	<0.001
SBP(mmHg)	0.126	0.007
DBP(mmHg)	0.061	0.190
BMI(kg/m^2^)	0.189	<0.001
WC(cm)	0.276	<0.001
TC(mmol/L)	0.008	0.870
TG*(mmol/L)	0.104	0.026
LDL-C(mmol/L)	0.015	0.752
HDL-C(mmol/L)	-0.121	0.009
CRE*(µmol/L)	0.332	<0.001
GFR(mL/min)	-0.165	<0.001
SUA(µmol/L)	0.622	<0.001
FPG(mmol/L)	-0.113	0.015
HbA1c(%)	-0.194	<0.001
AST*(U/L)	0.205	<0.001
ALT*(U/L)	0.144	<0.001
25-hydroxy vitamin D	-0.122	0.009

Continuous data are presented as Pearson correlation coefficient (r). Skewed distribution data are expressed as median (25th, 75th) percentiles.

SBP, Systolic Blood Pressure; DBP, Diastolic Blood Pressure; BMI, Body Mass Index; WC, Waist Circumference; TC, Total Cholesterol; TG, Triglycerides; LDL-C, Low-Density Lipoprotein Cholesterol; HDL-C, High-Density Lipoprotein Cholesterol; CRE, Serum Creatinine; GFR, Glomerular Filtration Rate; SUA, Serum Uric Acid; FPG, Fasting Plasma Glucose; HbA1c, Glycated Hemoglobin; AST, Aspartate Aminotransferase; ALT, Alanine Aminotransferase.

### The results of multiple stepwise linear regression analysis of serum asprosin level and related indexes

Utilizing multiple stepwise linear regression analysis with serum asprosin as the dependent variable and age, BMI, SBP, HbA1c, creatinine, LDL-C, 25-hydroxy vitamin D, and SUA as independent variables, the study found that age, 25-hydroxy vitamin D, SUA, creatinine, and LDL-C were independent influencing factors for serum asprosin (P < 0.05, **Table 3**).

**Table 3:** Multiple stepwise linear regression analysis of factors influencing serum asprosin in patients with type 2 diabetes.

independent variable	β value	SE value	β’	t value	*P value*	95%CI
age	0.956	0.228	0.137	4.187	0.000	0.507~1.404
25-hydroxyvitamin D	-0.932	0.202	-0.149	-4.626	0.000	-1.328~-0.536
SUA	0.686	0.036	0.660	19.251	0.000	0.616~0.757
creatinine	0.394	0.158	0.086	2.488	0.013	0.083~0.705
LDL-C	7.053	3.308	0.070	2.132	0.034	0553~13.553

LDL-C, Low-Density Lipoprotein Cholesterol; CRE, Serum Creatinine; SUA, Serum Uric Acid; 25-hydroxyvitamin D.

### Correlation analysis of 25-hydroxy vitamin D and serum asprosin levels indifferent groups

**Figure 1:**
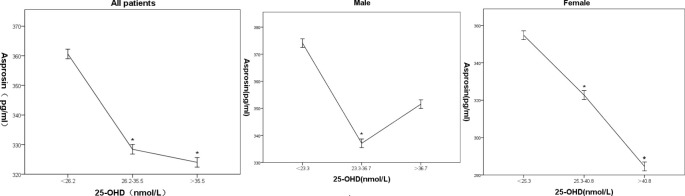
Association of 25-hydroxy Vitamin D with Serum Asprosin in Patients with Type 2 Diabetes in all, male and female patients. Mean serum asprosin and 95% confidence intervals corresponding to three quantiles of 25-hydroxyvitamin D; * indicates statistical significance compared to the first quantile array (*P < 0.05).

By grouping patients into three quantiles of 25-hydroxy vitamin D (All patients: T1 < 26.2 nmol/L, T2 26.2~35.5 nmol/L, T3 > 35.5 nmol/L; Male: T1 < 263.3nmol/L, T2 23.3~36.7 nmol/L, T3 > 36.7 nmol/L;Female:T1 < 25.3 nmol/L, T2 25.3~40.8 nmol/L, T3 > 40.8 nmol/L);, a general linear regression model was used with serum asprosin as the dependent variable. Mean serum asprosin levels (95% CI) for T1, T2, and T3 were 360.6 (346.2, 375.5), 328.4 (315.5, 341.3), and 324.0 (309.8, 338.2) in all patients, Mean serum asprosin levels (95% CI) for T1, T2, and T3 were 374.1 (352.1, 396.0, 337.1 (323.4, 350.8), and 351.6 (330.5 372.6) in male patients, Mean serum asprosin levels (95% CI) for T1, T2, and T3 were 354.8 (324.2, 385.4), 322.8 (306.6, 338.9), and 284.6 (259.1, 309.9) in female patients, respectively. Compared to the T1 group, serum asprosin levels were significantly reduced in the other two groups between all patients and female patients (P < 0.01). As 25-hydroxy vitamin D levels increased from T1 to T3, mean serum asprosin gradually decreased (**Figure 1**). Compared to T2 groups, serum asprosin levels were significantly increased in male patients (**Figure 1**).

## Discussion

The findings of this study demonstrated that in community-dwelling patients with type 2 diabetes, there was a gradual increase in age, disease duration, SBP, BMI, WC, creatinine, and SUA levels with the rise in serum asprosin levels. Conversely, serum asprosin displayed an inverse correlation with 25-hydroxy vitamin D levels, indicating a gradual decrease in serum asprosin as 25-hydroxy vitamin D levels increased.

25-hydroxy vitamin D deficiency has been identified as a common underlying factor for metabolic disorders like obesity, type 2 diabetes, and hypertension (9–12). Asprosin, a novel protein hormone found in white adipose tissue, has been shown in animal studies to exhibit elevated serum levels in mammals with insulin resistance (1, 13). In population-based investigations involving polycystic ovary syndrome patients and women with type 2 diabetes, higher serum asprosin levels were identified compared to healthy individuals (14). The present study revealed that heightened serum asprosin levels were associated with indicators linked to insulin resistance, including elevated BMI and WC levels, as well as decreased blood glucose and 25-hydroxy vitamin D levels. Correlation analysis further indicated a positive association between serum asprosin and BMI, as well as TG, while a negative correlation existed between serum asprosin and 25-hydroxy vitamin D. These findings suggest a potential connection between increased serum asprosin levels, decreased 25-hydroxy vitamin D, and insulin resistance in community-based patients with type 2 diabetes. Notably, patients with serum asprosin > 370.5 pg/ml displayed a higher proportion receiving oral hypoglycemic drugs and insulin-lowering therapy, possibly contributing to lower blood glucose levels, which aligns with findings by Wang (15).

The potential mechanism underlying the link between 25-hydroxy vitamin D and asprosin can be understood as follows (16–21). Firstly, Adipokines and adipose tissue may be a direct target for vitamin D. The expression of both the vitamin D receptor and 25-hydroxyvitamin D 1α-hydroxylase (CYP27B1) genes has been demonstrated in murine and human adipocytes. Secondly, Vitamin D metabolites might influence an increased asprosin production. Furthermore, Vitamin D may also be involved in the regulation of asprosin levels through modification of insulin sensitivity. The study’s findings revealed a negative correlation between serum asprosin and 25-hydroxy vitamin D levels in patients with type 2 diabetes, but this correlation was observed only within a certain range of 25-hydroxy vitamin D levels. The underlying cause remains uncertain and necessitates confirmation through further investigations. One hypothesis is that low stimulation of fat cells by 25-hydroxy vitamin D might contribute to conditions like obesity and insulin resistance. This could lead to elevated inflammatory factors like tumor necrosis factor-α (TNF-α) and interleukin-1 (IL-1), ultimately causing an imbalance in adipocytokine levels (22).

Type 2 diabetes and obesity have a comorbidity mechanism, which is related to 25 hydroxyvitamin D deficiency, and may affect the synthesis and metabolism of 25 hydroxyvitamin D through inflammation and oxidative stress. White adipose tissue is mainly produced by white adipocytes, and we speculate that obesity may indirectly affect the level of 25 hydroxyvitamin D by affecting the production of white adipose tissue.

Several limitations should be acknowledged in this study. Firstly, geographic factors, sample size, and population characteristics could have introduced confounding influences, and there might be unaccounted variables. Additionally, this study has a cross-sectional design, the relationship between 25-hydroxy vitamin D and serum asprosin might be bidirectional and interacting, necessitating further confirmation through prospective studies. Therefore, causal relationships between 25-hydroxy vitamin D and serum asprosin cannot be established solely from this study. Secondly, the assessment of islet function and insulin resistance index was lacking in this community-based study of patients with type 2 diabetes. Lastly, diet control and sunlight conditions during the research period were not evaluated in this study, so the effect on asprosin could not be more clearly reflected, which is also a limitation of this study”.

In addition, we found that with an increase in 25-OHD levels, the trend of serum asprosin levels in males and females was different. Currently, there are no reports on 25-OHD and asprosin levels, and we believe it may be related to changes in estrogen levels. Further confirmation is needed in other studies to determine whether there is a gender difference in the effect of 25-OHD on asprosin levels.

In conclusion, further investigation is warranted to understand the intricate regulatory role of 25-hydroxy vitamin D on adipocytokines, particularly its potential variations between health and disease states. This study underscored an inverse correlation between serum asprosin and 25-hydroxy vitamin D levels among patients with type 2 diabetes in the community.

## Data availability statement

The original contributions presented in the study are included in the article/supplementary material. Further inquiries can be directed to the corresponding authors.

## Ethics statement

The studies involving humans were approved by the Ethics Committee of the First Hospital of Shanxi Medical University (Approval number: 2019 [K056]). The studies were conducted in accordance with the local legislation and institutional requirements. The participants provided their written informed consent to participate in this study.

## Author contributions

JC: Conceptualization, Formal analysis, Methodology, Software, Validation, Writing – original draft. ZW: Data curation, Formal analysis, Methodology, Software, Writing – original draft. JYi: Data curation, Writing – original draft. MLi: Methodology, Writing – original draft. QW: Investigation, Writing – original draft. MLiu: Investigation, Writing – original draft. HS: Validation, Writing – original draft. HR: Visualization, Writing – original draft. MX: Investigation, Writing – original draft. JYa: Funding acquisition, Supervision, Writing – review & editing. LX: Conceptualization, Funding acquisition, Methodology, Project administration, Supervision, Writing – review & editing.
